# Long-Term Desynchronization by Coordinated Reset Stimulation in a Neural Network Model With Synaptic and Structural Plasticity

**DOI:** 10.3389/fphys.2021.716556

**Published:** 2021-09-08

**Authors:** Thanos Manos, Sandra Diaz-Pier, Peter A. Tass

**Affiliations:** ^1^Institute of Neuroscience and Medicine, Brain and Behaviour (INM-7), Research Centre Jülich, Jülich, Germany; ^2^Medical Faculty, Institute of Systems Neuroscience, Heinrich Heine University Düsseldorf, Düsseldorf, Germany; ^3^Laboratoire de Physique Théorique et Modélisation, CNRS, UMR 8089, CY Cergy Paris Université, Cergy-Pontoise Cedex, France; ^4^Simulation & Data Lab Neuroscience, Institute for Advanced Simulation, Jülich Supercomputing Centre (JSC), Forschungszentrum Jülich GmbH, JARA, Jülich, Germany; ^5^Department of Neurosurgery, Stanford University School of Medicine, Stanford, CA, United States

**Keywords:** coordinated reset neuromodulation, desynchronization, spike time-dependent plasticity, structural plasticity, anti-kindling

## Abstract

Several brain disorders are characterized by abnormal neuronal synchronization. To specifically counteract abnormal neuronal synchrony and, hence, related symptoms, coordinated reset (CR) stimulation was computationally developed. In principle, successive epochs of synchronizing and desynchronizing stimulation may reversibly move neural networks with plastic synapses back and forth between stable regimes with synchronized and desynchronized firing. Computationally derived predictions have been verified in pre-clinical and clinical studies, paving the way for novel therapies. However, as yet, computational models were not able to reproduce the clinically observed increase of desynchronizing effects of regularly administered CR stimulation intermingled by long stimulation-free epochs. We show that this clinically important phenomenon can be computationally reproduced by taking into account structural plasticity (SP), a mechanism that deletes or generates synapses in order to homeostatically adapt the firing rates of neurons to a set point-like target firing rate in the course of days to months. If we assume that CR stimulation favorably reduces the target firing rate of SP, the desynchronizing effects of CR stimulation increase after long stimulation-free epochs, in accordance with clinically observed phenomena. Our study highlights the pivotal role of stimulation- and dosing-induced modulation of homeostatic set points in therapeutic processes.

## Introduction

High-frequency deep brain stimulation (HF DBS) is the standard treatment of medically refractory movement disorders such as Parkinson’s disease (PD) (Benabid et al., 1991; Krack et al., 2003; Deuschl et al., 2006) and is also being tested in other psychiatric diseases (Cleary et al., 2015). HF DBS aims to permanently deliver electrical charge-balanced pulses at high frequencies (>100 Hz) to target areas such as the thalamic ventralis intermedius (VIM) nucleus or the subthalamic nucleus (STN) through chronically implanted depth electrodes (Benabid et al., 1991, 2009). However, the precise mechanism of action of standard HF DBS is not yet sufficiently understood (Johnson et al., 2008; Gradinaru et al., 2009; Deniau et al., 2010). Standard HF DBS has acute clinical (Temperli et al., 2003) and electrophysiological (Kühn et al., 2008) effects, which are present only during stimulation. A number of reversible as well as non-reversible adverse events (AE) can arise from DBS treatment, which is itself an invasive treatment; these can range from neurological AEs such as gait disturbances and speech problems, to psychiatric AEs such as depression (Baizabal-Carvallo and Jankovic, 2016; Buhmann et al., 2017). Therefore, any DBS treatment regime should be minimized in duration as much as possible, in order to also minimize the AEs that can result (Buhmann et al., 2017).

Abnormal neuronal synchronization is a hallmark of PD and may occur in different frequency ranges (Lenz et al., 1994; Nini et al., 1995; Llinás et al., 1999; Brown, 2002). Different stimulation protocols showed that trains of HF DBS may in fact modulate synaptic plasticity (Shen et al., 2003; Prescott et al., 2009; Milosevic et al., 2018). There are several different mechanisms of synaptic plasticity, of which spike-timing-dependent plasticity (STDP) is the most well-known and widely accepted (see, e.g., Lisman, 2017). STDP is a fundamental synaptic plasticity mechanism which modifies synaptic strengths based on the relative timing of pre- and postsynaptic spike pairs (Gerstner et al., 1996; Markram et al., 1997; Bi and Poo, 1998, 2001; Song et al., 2000). According to the STDP mechanism, the synaptic strength is potentiated when the postsynaptic spike follows the presynaptic spike; conversely, the synaptic strength is depressed when the postsynaptic spike advances the presynaptic spike (Markram et al., 1997).

Coordinated reset (CR) stimulation is a computationally developed patterned multichannel stimulation (Tass, 2003a,b) that employs STDP to induce long-lasting therapeutic effects (Tass and Majtanik, 2006). CR stimulation specifically counteracts abnormal neuronal synchrony by desynchronization (Tass, 2003a,b), which causes a reduction of the rate of coincidences and, mediated by STDP, a reduction of the synaptic weights (Tass and Majtanik, 2006). Consequently, as shown computationally, neuronal networks are shifted from unfavorable attractors (with strong neuronal synchrony and synaptic connectivity) to more favorable attractors (with weak synchrony and reduced synaptic weights) (Tass and Majtanik, 2006). Accordingly, in neuronal network models a long-lasting desynchronization can be achieved. The term long-lasting desynchronization refers to desynchronization which exceeds cessation of stimulation (see Tass and Majtanik, 2006; Manos et al., 2018a; Kromer and Tass, 2020). Such effects have been demonstrated pre-clinically and clinically in a variety of studies (e.g., Tass et al., 2009, 2012a,b; Adamchic et al., 2012, 2013; Pfeifer et al., 2021). For CR-DBS delivered to the STN, computationally predicted desynchronizing effects (Tass, 2003a,b), cumulative effects (Hauptmann and Tass, 2009) and long-lasting effects (Tass and Majtanik, 2006) were verified in pre-clinical studies in a 1-methyl-4-phenyl-1,2,3,6-tetrahydropyridine (MPTP) monkey PD model (Tass et al., 2012b; Wang et al., 2016) as well as in a proof-of-concept study in PD patients (Adamchic et al., 2014a).

In accordance with computational predictions (Popovych and Tass, 2012; Tass and Popovych, 2012), CR stimulation can also be realized by means of sensory stimulation modalities and induce long-lasting desynchronization. In a proof-of-concept study in patients with chronic subjective tinnitus, acoustic CR stimulation caused a significant and sustained reduction of tinnitus symptoms (Tass et al., 2012a) combined with a significant reduction of abnormal neuronal synchrony (Adamchic et al., 2012, 2013; Tass et al., 2012a), abnormal cross-frequency coupling (Adamchic et al., 2014b) and abnormal effective connectivity (Silchenko et al., 2013). In a study of PD patients, vibrotactile CR (vCR) stimulation (Tass, 2017) delivered to the fingertips during three consecutive days for 4 h per day caused improvements in gait and bradykinesia for up to one month post-stimulation (Syrkin-Nikolau et al., 2018). A case series in three PD patients treated with daily regular or noisy vCR fingertip stimulation during 6+ months revealed continuous improvement of PD motor symptoms as assessed after medication withdrawal (i.e., off medication) (Pfeifer et al., 2021). Regular vCR uses periodic stimulus delivery, whereas for noisy vCR stimulus onsets had a moderate jitter in time (Pfeifer et al., 2021). In addition, in a pilot study six PD patients received daily noisy vCR stimulation during 3 months (Pfeifer et al., 2021). Motor evaluations and at-rest electroencephalographic (EEG) recordings, performed off medication before and after the 3-month vCR therapy, revealed a statistically and clinically significant motor improvement along with a significant decrease of cortical sensorimotor high beta power (21–30 Hz). Both studies with sensory CR performed so far (Tass et al., 2012a; Pfeifer et al., 2021) provide preliminary evidence indicating that the effects of repetitively administered acoustic as well as vibrotactile CR stimulation may increase in the course of the treatment, in this way enabling longer treatment pauses and reduced dosage regimens. However, in the computational studies performed as yet, memory-type effects, mediating an increase of CR efficacy with repetitive stimulation delivery, have not been described. We hypothesize that structural plasticity, acting on a slower time scale than synaptic plasticity (Fauth and Tetzlaff, 2016), might play a role in modifying CR effects in the course of repeated delivery of CR stimuli.

Apart from synaptic plasticity, structural plasticity also appears to play an important role in PD. Structural plasticity allows neurons to establish new or delete pre-existing synaptic connections; this occurs via the extension or retraction of axons and dendrites, or by modifying the number of axonal boutons or dendritic spines (Butz et al., 2009). Changes in structural connectivity and rewiring of connections is crucial for memory formation (Xu et al., 2009) as well as for adaptive and maladaptive processes in response to central and peripheral lesions of the nervous system (Keck et al., 2008; Yamahachi et al., 2009; Brown et al., 2010) and neurodegeneration (Deller et al., 2006). A study of the gray matter value in the basal ganglia (BG) of patients with symptomatic PD revealed that BG undergo a form of progressive atrophy that increases with disease duration and severity (Reetz et al., 2009). Furthermore, it was shown that balance training induces morphometric changes in PD patients (Sehm et al., 2014): a voxel-based morphometry revealed performance improvement-correlated gray matter changes in the right anterior precuneus, left inferior parietal cortex, left ventral premotor cortex, bilateral anterior cingulate cortex, and left middle temporal gyrus as well as time-dependent gray matter changes in the right cerebellum. In contrast, only learning-dependent gray matter changes in the left hippocampus were observed in healthy controls (Sehm et al., 2014). In a single PD patient case study, it was shown that long-term HF DBS gave rise to significant localized structural changes in sensory-motor, prefrontal, limbic, and olfactory brain regions together with a restoration of functional connectivity (van Hartevelt et al., 2014). In addition, a computational analysis revealed that the observed changes of structural weights are compliant with a hypothesized Hebbian-like mechanism (van Hartevelt et al., 2015). For a more extensive discussion on the role of structural plasticity in the brain please see the corresponding section of the Supplementary Material and van Ooyen and Butz-Ostendorf (2017).

Accordingly, we set out to computationally integrate a structural plasticity mechanism into our modeling approach; thus, in addition to STDP we also used the built-in structural plasticity module (Diaz-Pier et al., 2016) of the NEST platform (Bos et al., 2015). NEST is a simulator for neuronal modeling, and one which can incorporate synaptic plasticity modules. Structural plasticity has two main areas of application in NEST. The primary purpose is to model the neurobiological phenomenon of morphological transformations that a neuron undergoes over time, leading to the creation or deletion of synapses. For a more complete description of the model see Butz and van Ooyen (2013) and for the details on the exact implementation see Diaz-Pier et al. (2016). Specifically, the algorithm serves a homeostatic purpose by self-generating or self-eliminating connections in order to reach and maintain the target firing rate of the network. New synapses are plastic, and their weight-values are drawn from the same probability distribution defined in the initialization of the simulation. In order to achieve this, the algorithm follows a set of homeostatic rules which dictate how the connectivity should be modified in order to achieve the desired levels of electrical activity. The algorithm works at the single neuron level, so that each neuron follows this rule independently. Contrary to STDP, the structural plasticity mechanism allows connections to be deleted and created between neurons, and not only alter their weights. By combining both plasticity mechanisms, we increased the sampling capacities and the plasticity potential of the system to explore different connectivity configurations, allowing long-term structural changes and short-term learning in the same simulation.

We hypothesize that structural plasticity may modify long-term effects of CR stimulation. Motivated by experimental findings indicating an increased efficacy of sensory CR stimulation in the course of the therapy (Tass et al., 2012a; Pfeifer et al., 2021), we incorporate structural plasticity in our computational model and consider different treatment scenarios, depending on whether CR stimulation may induce changes of the set point of structural plasticity. The efficacy of CR stimulation after long pauses without stimulation may strongly increase provided CR stimulation causes a reduction of the set point. The computational approach presented here may provide predictions that contribute to optimized and experimentally testable dosage regimens, which specifically aim at modulating homeostatic set points of structural plasticity mechanisms.

## Materials and Methods

### The Terman-Rubin Neuron Model

The Terman and Rubin (Terman et al., 2002; Rubin and Terman, 2004) single-compartment conductance-based model is used for the description of the neuronal activity of the individual STN and globus pallidus external (GPe) neurons. The membrane potential is given by the following equation:

(1) $$c_{\text{m}}\frac{\text{d}v}{\text{d}t}=-I_{\text{L}}-I_{\text{K}}-I_{\text{Na}}-I_{\text{T}}-I_{\text{Ca}}-I_{\text{ahp}}-I_{\text{syn}}+I_{\text{stim}}+I_{\text{noise}}$$

Spiking activity is caused by the sodium (Na^+^) and potassium (K^+^) ionic currents *I*_Na_, *I*_K_, *I*_*T*_, and *I*_*Ca *_describe the low-threshold T-type and high-threshold Ca^2+^ current, respectively. *I*_*ahp*_ represents a Ca^2+^-activated, voltage-independent after-hyperpolarization K^+^ current and *I*_L_ the leak current. In addition, the STN and GPe neurons are influenced by synaptic inputs *I*_*syn*_. Surrounding brain areas contribute with *I*_*noise*_ while there is an external stimulation current *I*_*stim*_ (only in the STN population) which models the deep brain stimulation (DBS). Other ionic currents (in pA/μm^2^) are described by Terman et al. (2002); Rubin and Terman (2004), and Ebert et al. (2014).

(2) $$I_{\text{L}}=g_{\text{L}}\left[v-v_{\text{L}}\right]$$

(3) $$I_{\text{K}}=g_{\text{K}}n^{4}\left[v-v_{\text{K}}\right]$$

(4) $$I_{T}=g_{\text{T}}a_{\infty }^{3}\left(v\right)b_{\infty }^{2}\left(r\right)\left[v-v_{\text{Ca}}\right]$$

(5) $$I_{\text{Ca}}=g_{\text{Ca}}s_{\infty }^{2}\left(v\right)h\left[v-v_{\text{Ca}}\right]$$

(6) $$I_{\text{ahp}}=g_{\text{ahp}}\left[v-v_{\text{K}}\right]\frac{\left[\text{Ca}\right]}{\left[\text{Ca}\right]+k_{1}}$$

(7) $$\frac{\text{d}\left[\text{Ca}\right]}{\text{d}t}=\text{Γ}\left(-I_{\text{Ca}}-I_{\text{T}}-k_{\text{Ca}}\left[\text{Ca}\right]\right)$$

Subthalamic nucleus and GPe neurons are described by similar equations and they differ only in a few parameter values (see Supplementary Tables 1, 2) as well as in the form of the low threshold Ca^2+^ current:

(8) $$I_{\text{T}}=g_{\text{T}}a_{\infty }^{3}\left(v\right)r\left[v-v_{\text{Ca}}\right]$$

for the GPe neurons (i.e., the $b_{\infty }^{2}$ term is omitted). Moreover, neurons are not identical, i.e., their reverse potential parameters and ion channel maximum conductances are drawn from Gaussian probability distributions with 10% standard deviation around the mean value (except the parameter *v*_*L*_ where we draw values with 0.15% standard deviation). All the mean parameter values and their units are summarized in Supplementary Tables 1, 2. The slowly operating gating variables *n, h*, and *r* depend on time and voltage and their first-order kinetics are governed by differential equations of the form:

(9) $$\frac{\text{d}X}{\text{d}t}=\varphi_{X}\left[\frac{X_{\infty }\left(v\right)-X}{\tau_{X}\left(v\right)}\right],$$

Where *X*:*n, h, r* with:

(10) $$\tau_{X}\left(v\right)=\tau_{X}^{0}+\frac{\tau_{X}^{1}}{1+\text{exp}\left[-\frac{v-\theta_{X}^{\tau }}{o_{X}^{\tau }}\right]},$$

where θ^τ^ is the voltage at which the time constant is midway between its maximum and minimum values, and *o*^τ^ is the slope factor for the voltage dependence of the time constant. Activation gating for the rapidly activating channels (*m, a*, and *s*) was treated as instantaneous. For all gating variables *X* = *n, m, h, a, r*, or *s*, the steady-state voltage dependence was determined using:

(11) $$X_{\infty }\left(v\right)=\frac{1}{1+\exp\left[-\frac{v-\theta_{X}}{o_{X}}\right]},$$

where θ_*X*_ is the half activation (or inactivation) voltage for gating variable *X*, and *o*_*X*_ is the slope factor for that variable. For the *T* current inactivation variable *b*, we used:

(12) $$b_{\infty }\left(r\right)=\frac{1}{1+\text{exp}\left[\frac{r-\theta_{b}}{o_{b}}\right]}-\frac{1}{1+\text{exp}\left[-\frac{\theta_{b}}{o_{b}}\right]},$$

See Terman et al. (2002) and Rubin and Terman (2004) for more information on the model and the variables’ units. One main difference of our study compared to Ebert et al. (2014) is the initialization of the STN synaptic weights. In Ebert et al. (2014), the initial mean synaptic weight was set at ${\bar{w}}_{\text{ss}}^{\text{STN}}$ = 0.018, in order to tune the system at a strongly synchronized state. Here, we chose a different approach. We set ${\bar{w}}_{\text{ss}}^{\text{STN}}$ = 0.0025, at a much lower value, and drove the system to a similar strongly synchronized state by applying periodic stimulation (PS) to the STN neurons.

In our NEST implementation, which is based on NEST 2.10, we have incorporated an additional feature which allows us to enable and disable synaptic and structural plasticity. We have also modified the *topology module* in order to allow plastic synapses to be created. The Terman-Rubin neuron model (STN and GPe populations) was implemented in the NESTML platform (Plotnikov et al., 2016; Blundell et al., 2018; Perun et al., 2018).

### The Network Model

**FIGURE 1:**
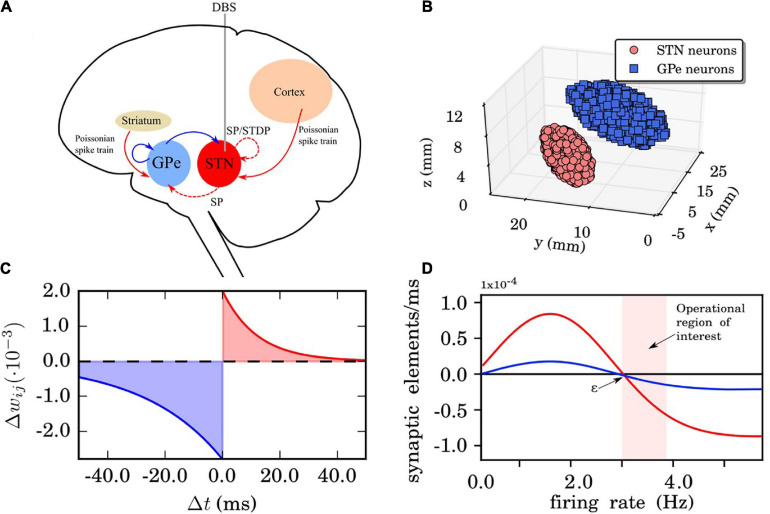
Network, synaptic, and structural plasticity setup. **(A)** Structure of the model network. Excitatory synaptic connections are shown in red (with arrows) and inhibitory connections in blue (with squares). The connections whose synaptic weights do not vary in time are in solid lines. The red dashed line represents time dependent connections between the subthalamic nucleus (STN) neurons, modified by spike time-dependent plasticity (STDP) and structural plasticity. The external electrical stimulation delivered to the STN is indicated by the gray solid line. The Poissonian spike train input from the cortex to the STN is constantly active. **(B)** The three-dimensional STN-GPe model (left brain hemisphere). Segmented MRI slices taken from a Parkinson’s disease (PD) patient prior to deep brain stimulation (DBS) surgery. Each ellipsoid volume contains 1,000 randomly distributed point-like neurons. Note, neurons are not overlapping. **(C)** STDP rule for STN neurons. Synaptic connection potentiation is highlighted in red and depression in blue. **(D)** Growth rate curves determining the rate of creation and deletion of synaptic elements in the structural plasticity model for STN-STN connections (red) and STN-GPe connections (blue). The parameters which define the shape of the curve are the growth rate ν_*SP*_, which defines the peak of the curve, and the target firing rate ε. The light red band indicates the region where the model operates during the simulations reported in this study. These Gaussian curves correspond to Eq. (19) which represents the change in synaptic elements *dz/dt* as a function of the firing rate FR(*t*). In this case, the red line has a growth rate ν_*SP*_ of 0.00008 synaptic elements/ms and the blue line of 0.00002 synaptic elements/ms. The target firing rate ε in both curves is 3.0 Hz. Finally, the minimal initial firing rate η is 0.0 for both curves.

Our network model consists of two interacting nuclei, the STN and the GPe. The STN neuron population is linked with the cortex in an excitatory manner while the GPe population is linked with the striatum in an inhibitory manner, see Figure 1A. The connectivity matrix of the STN-GPe network can be expressed as the combination of several sub-networks:

(13) $$W=\left(\begin{array}{cc} w_{\text{ss}} & w_{gs}\\ w_{\text{sg}} & w_{\text{gg}} \end{array}\right)$$

Denoting the connectivity matrices for the STN-STN (ss), STN-GPe (sg), GPe-STN (gs), and GPe-GPe (gg) sub-networks, respectively. Each *W*_*ij*_ value corresponds to the connection strength between neuron *i* and *j* while self-connections are excluded, i.e., *W*_*ii*_ = 0,∀*i*. Our neuronal network is a weighted and directed graph, and hence, this matrix is not necessarily symmetric.

The STN population projects long range excitatory connections on the GPe population, while the GPe population projects long range inhibitory connections to the STN neurons (Shink et al., 1996). The connections between STN and GPe were established following Baufreton et al. (2009) and using connectivity values of 2% (i.e., 200 synapses per neuron, connecting to 20 randomly chosen targets in the distant population, respectively). Following Fujimoto and Kita (1993) and Ebert et al. (2014), the transmission delay for connections between the STN and the GPe was set to δ_sg_ = δ_gs_ = 4.0*ms*. Recent findings on the synaptic organization of the STN population identifying its neurons as parallel processing units (Steiner et al., 2019) might help to further refine computational models of the BG.

### Network and Neuron Coupling Description

The network setup and 3D anatomical arrangement of the neurons was introduced in Ebert et al. (2014) where a sample of 10,000 STN and 10,000 GPe neuron coordinates was drawn. The coordinates used for the 3D implementation originated from magnetic resonance imaging (MRI) data taken from a PD patient before DBS surgery (both STN and GPe coordinates are measured in the left-brain hemisphere while there is no actual overlap of the single neurons, Figure 1B). The rationale for choosing equal size (# of neurons) for both populations (even though the GPe area is larger than STN) is that the GPe has a lower density of neurons than the STN (see Levesque and Parent, 2005). The two populations form two ellipsoids of different size, inside of which coordinates for the neuron positions are randomly drawn. For more details on the 3D implementation with two set of coordinates and ellipsoid dimensions and its motivation, we refer to Ebert et al. (2014).

The general connectivity properties are largely based on the values used and presented in Ebert et al. (2014), where 10,000 STN and 10,000 GPe neurons were considered [more details regarding the parameter values can also be found in Gillies and Willshaw (1998) and Holgado et al. (2010)]. The main difference in this study is that we have considered a smaller in size neural network (1,000 STN and 1,000 GPe neurons instead of 2×10,000) in order to trade-off between a large-scale model and the corresponding computation time. Hence, the each STN neuron extends connections to *p*_ss_ = 70% of the entire STN population (instead of *p*_ss_ = 7% that was used for the large-scale system). The connection probability between STN neurons at a certain distance is given by the exponential function *p*(*x*) = e^−*x*/*c*_d_^ with *c*_*d*_ = 0.5 and *x* the Euclidean distance between neurons. This probability is a non-negative function with values in the interval [0, 1]. Note, it is not a probability density which would have an integral equal to 1. The corresponding parameter values for the inhibitory GPe neurons are *p*_gg_ = 10% (instead of *p*_gg_ = 1% that was used for the large-scale system),δ_gg_ =  4.0 ms and c_d_ = 0.63. The initial synaptic weights for both STN and GPe connections are drawn from a Gaussian probability distribution around a mean value ${\bar{w}}_{\text{ss}}^{\text{STN}}=0.0025$ with standard deviation $\sigma_{\text{ss}}^{\text{STN}}=0.000125$ (no distance dependence). The two structures are connected to each other as the STN affects GPe via excitatory input while the GPe exerts an inhibitory impact on the STN. There is no distance dependence for their connectivity. We adopted a probability connectivity (see Baufreton et al., 2009) of 20% (instead of 2% that was used for the large-scale system), i.e., 200 fixed randomly picked synapses with transmission delay of δ_sg_ = δ_gs_ = 6.0ms for connections between the STN and the GPe, as published in Fujimoto and Kita (1993) and Kita et al. (2005). Their corresponding initial synaptic weights are drawn again from a Gaussian probability distribution around a mean value ${\bar{w}}_{\text{sg}}^{\text{STN}}=0.006$ and ${\bar{w}}_{\text{gs}}^{\text{STN}}=0.003$ with standard deviation $\sigma_{\text{sg}}^{\text{STN}}=0.0003$ and $\sigma_{\text{sg}}^{\text{STN}}=0.00015$, respectively.

Using the aforementioned described parameter values, we achieved a good agreement between the two networks of different size concerning their dynamics, as assessed by macroscopic quantities, degree of synchrony (as measured by the order parameter throughout this study) and mean firing rate, we adjusted all relative connections in a similar manner. The comparison between the large network (2 × 10,000 neurons) with the reduced size one (2 × 1,000) can be found in Supplementary Figure 1 showing similar overall system evolution without any significant qualitative differences.

### Synaptic Currents

The postsynaptic currents are described with α -functions (Dayan and Abbott, 2000; Gerstner and Kistler, 2002):

(14) $$\alpha \left(t\right)=\frac{t-t_{k}}{\tau_{\text{syn}}^{2}}{\text{e}}^{-\frac{t-t_{k}}{\tau_{\text{syn}}}},t_{k}\leq t<t_{k+1}$$

Where *t*_*k*_ denotes the spike time. The total synaptic input current to a postsynaptic neuron *i* received from presynaptic neurons *j* is then given by:

(15) $$I_{\text{syn},i}\left(t\right)=\sum_{j}W_{ij}\left(v_{i}\left(t\right)-v_{\text{syn}}\right)\alpha \left(t\right),$$

Where *W*_*ij*_ is the synaptic weight (coupling strength) between presynaptic neurons *j* and the postsynaptic neuron *i*, and *v*_*syn*_ is the reversal potential for excitatory or inhibitory ss, sg, gs, and gg connections. Therefore, *v*_*syn*_ depends on the types of connected neurons and on whether the connection is excitatory or inhibitory. In our model, there exist four types of synapses for ss, sg, gs, and gg connections. All values are given in Supplementary Tables 1, 2.

### Noise Inputs

Each GPe neuron receives external inhibitory input from the striatum which is described by an additional constant negative input current *I*_app_ = −7.0pAin Eq. (1) (note that for the excitatory STN neurons *I*_app_ =  0pA) and external noise *I*_*noise*_ described by Poissonian spike trains (form other surrounding brain structures) with a frequency of ${\text{f}}_{\text{P}}^{\text{GPe}}=40\text{Hz}$:

(16) $$I_{\text{noise},i}=\sum_{j=0}^{N_{t}}w_{\text{noise}}\left(v_{j,i}\left(t\right)-v_{\text{noise}}\right)\alpha \left(t\right).$$

*N*_*t*_ is an integer random variable following the Poisson distribution $P_{\lambda }\left(k\right)=\frac{\lambda^{k}}{k!}{\text{e}}^{-\lambda }$, with parameter given by the simulated time interval λ=*f*_*P*_δ, where δ is equal to the time resolution of the simulation (0.1 ms in our simulations). In a similar manner, we model the excitatory input from the cortex to all STN neurons with a frequency of ${\text{f}}_{\text{P}}^{\text{GPe}}=20\text{Hz}$. Each neuron receives a different random Poissonian spike train. These noise frequency values are chosen with respect to the respective STN and GPe relative firing rates, i.e., the mean firing rate of GPe is about twice the one of the STN [see Ebert et al. (2014) and references therein for motivation and justification]. The noise input synaptic weight is set at *w*_noise_ =  0.2mS/cm^2^ while the time constants for the α -function are set at τ_noise_ = 1.0 ms and the reversal potential is *v*_noise_ = 0mV for both STN and GPe neurons.

### Spike-Timing-Dependent Plasticity

The synaptic coupling weights (*w*_*ij*_) of the STN neurons evolve dynamically and depend on the time difference ($\Delta t_{ij}=t_{j}^{f}-t_{i}^{f}$) between the firing (onset) of the spikes of the post-synaptic neuron and pre-synaptic neuron (denoted by $t_{i}^{f}$ and $t_{j}^{f}$, respectively). When Δ*t*_*ij*_ > 0 the rule implements synaptic potentiation due to causal relationships and when Δ*t*_*ij*_ < 0 it implements synaptic depression. We use the following STDP rule as implemented in NEST:

(17) $$\Delta w_{ij}\left(\Delta t_{ij}\right)=\left\{ \begin{array}{c} \lambda e^{-\frac{\left|\Delta t_{ij}\right|}{\tau_{+}}},\Delta t_{ij}>0\\ -\lambda \gamma e^{-\frac{\left|\Delta t_{ij}\right|}{\tau_{-}}},\Delta t_{ij}\leq 0 \end{array}\right.,$$

where τ_−_ =  27.5ms,τ_+_ =  12.0ms (time constants for the synaptic weight change of depression and potentiation, respectively),λ =  0.002(learning rate of the synaptic connection) andγ = 1.4 is the ratio between depression and potentiation in the synaptic learning rule (Figure 1C). The (de)synchronized dynamics are stable with the above rule and parameter values resulting in multistability. We restrict the synaptic weights $w_{ij}^{\text{ss}}$ (within the STN population) on the interval[0,0.02], in this way avoiding a non-physiological unbounded increase or decrease. More detailed analysis and parameter exploration of this particular STPD rule and its motivation can also be found in Ebert et al. (2014).

### Structural Plasticity

Using the structural plasticity framework in NEST, a network will self-generate synapses in order to stably achieve a desired profile of electrical activity, a measure that is experimentally more easily accessible than detailed connectivity data. By progressively and slowly changing the connections between neurons in the network and the weight of these connections for all populations simultaneously, the structural plasticity algorithm is able to find a stable configuration with the desired firing rate profile. The structural plasticity implementation in NEST is based on the model proposed by Butz and van Ooyen (2013) and described in technical detail by Diaz-Pier et al. (2016). In this plasticity framework, neurons have contact points called synaptic elements which increase or decrease in number according to simple homeostatic rules. This rule is originally based on the intracellular calcium concentration (Butz and van Ooyen, 2013; Diaz-Pier et al., 2016) which is modulated by the network’s firing rate. Synaptic elements typically represent axonal boutons and dendritic spines. When new synaptic elements become available, they can be used to create new synapses with other compatible elements. If the contact points are eliminated, the synapses formed earlier are destroyed. Connectivity in the network is updated on a much slower timescale than the electrical activity of neurons. In order to have a computationally efficient simulation, connectivity in the network is updated between 100 and 1,000 times more slowly than the electrical activity in the neurons. The homeostatic rules applied to the synaptic elements are intended to keep the mean firing rate stable. The firing rate FR(*t*) is calculated in NEST by each neuron following this equation:

(18) $$\frac{\text{dFR}}{\text{d}t}=\left\{ \begin{array}{c} -\frac{\text{FR}\left(t\right)}{\tau_{\text{SP}}}+\beta ,\mathrm{if}\mathrm{the}\text{neuron}\mathrm{fires}\\ -\frac{\text{FR}\left(t\right)}{\tau_{\text{SP}}},\mathrm{otherwise} \end{array}\right.,$$

FR is the firing rate of the neuron which is directly proportional to the internal calcium concentration of the neuron at any point in time (Diaz-Pier et al., 2016), β is the calcium intake constant which specifies the increment of the internal calcium concentration every time the neuron spikes and τ_SP_ is the calcium concentration decay time constant. This means that the firing rate is internally calculated by NEST for each neuron model using a low pass filtering technique on the spiking activity corresponding to the instantaneous mean value of the firing rate. For more details on how this calculation is performed as well as the impact of the update interval and other structural plasticity parameters in the evolution of the simulations see Nowke et al. (2018). The firing rate is always calculated by NEST, even when structural plasticity is not enabled. In NEST it is possible to use different types of growth curves for the synaptic elements like Linear, Gaussian, and Sigmoidal. Additionally, users can implement their own growth curves to match their specific questions. In the current study, a Gaussian curve as defined in Figure 1D describes the growth rate of connection points for neurons, i.e.:

(19) $$\frac{\text{d}z}{\text{d}t}=\nu_{\text{SP}}\left[2{\text{e}}^{-{\left(\frac{\text{FR}\left(t\right)-\text{ξ}}{\zeta }\right)}^{2}}-1\right],$$

where *z* is the number of synaptic elements, $\text{ξ}=\frac{\eta +\varepsilon }{2}$, $\zeta =\frac{\varepsilon -\eta }{2\sqrt{ln2}}$, the maximum amplitude is the growth rate ν_*SP*_, the target firing rate ε is the right intersection with the *x*-axis and the minimum firing rate η is the left intersection with the *x*-axis. In our application there are no negative firing rates and η is omitted from Figure 1D as we are only interested in the curve on the positive side of the *x*-axis. Internally, each neuron has a count of the number of connected synaptic elements (*z*_connected). When z is higher than *z*_connected, new connections can be made and when *z* is lower than *z*_connected, some connections must be deleted. This will be done until *z* = z_connected for each update in connectivity. For an analysis of the dynamical stability caused by structural changes induced by structural plasticity in other network models we refer to Nowke et al. (2018).

Spike time-dependent plasticity and structural plasticity act on very different time scales. Let us consider the time scale if only structural plasticity is turned ON, whereas STDP is turned OFF. The growth rate of the structural plasticity mechanism used in our simulations reflects a timescale of network formation which corresponds to a timescale of days or weeks (Butz and van Ooyen, 2013; Butz et al., 2014). Note, arbitrary units were introduced in the structural plasticity model mechanism because there is no exact experimental information about the speed of creation or deletion of synapses in the brain regions of interest available (Butz and van Ooyen, 2013; Butz et al., 2014). There are experimental studies showing that the time it takes for boutons and spines to form ranges from days to weeks, as shown, e.g., in the context of early development (Kirov et al., 2004). The structural plasticity model is not dimensionless in time (Butz and van Ooyen, 2013; Butz et al., 2014). However, due to the lack of detailed experimental data the growth rate is not adapted to experimentally pre-defined values. Rather, the growth rate in this model of structural plasticity can be used to change the speed at which effects in the structure of the network are visible and it is chosen so that the total duration of the simulations, with time-steps in milliseconds, do not require to sum up to weeks, otherwise they would be computationally intractable. In our simulations we have shown 20 min of simulation which do not represent 20 min of biological time but a timescale of days or weeks. However, the massive reduction of computation time for the structural plasticity-induced dynamics is only possible if STDP is turned OFF while structural plasticity is turned ON (see below). Due to the pronounced structural changes induced in the network by SP, it is necessary to split the simulation and keep the STDP part separated from the SP part, in order to ensure numerical accuracy in the results.

### Coordinated Reset Stimulation

In order to model the invasive DBS electrical CR stimulation delivered to the STN population, we consider short biphasic current-controlled pulses as previously used in Ebert et al. (2014) [see Cogan (2008) for original motivation]. In more detail, a current pulse *P*(*t*) for stimulation of neuronal tissue consists of a cathodal and an anodal phase with current amplitudes and durations that result in an overall zero net charge for the entire pulse (to guarantee charge-balance):

(20) $$P\left(t\right)=\{\begin{array}{c} \kappa ,t_{l}\leq t<t_{l}+\omega \\ -\kappa /p_{s},t_{l}+\omega \leq t<t_{l}+\omega \left(1+p_{s}\right).\\ 0,\text{else} \end{array}$$

*t*_*l*_ denotes the onset times of the current pulses, κ is the amplitude and ω is the width of the cathodal pulse. *p*_*s*_ determines the duration and amplitude of the charge-balancing anodal pulse part which prevents any permanent charge transfer into the neuronal tissue that could possibly damage the tissue. In this study, we used a fixed electrode position at the center of the STN population which was found to be most adequate for optimal CR stimulation performance in Ebert et al. (2014). In order to model the electric field produced by the external stimulus, we used the setup of a *Medtronic DBS lead model 3389 which has* four separate cylindrical contacts made of a Pt-Ir alloy with a typical length of 1.5 mm (Coffey, 2009). We used a specific equation which adequately approximates the overall distance dependent decay of stimulation strength as used in Ebert et al. (2014; see also Richardson et al., 2003):

(21) $$S\left(d_{il}\right)=\frac{1}{d_{il}l_{c}\sqrt{1+4{\left(d_{il}/l_{c}\right)}^{2}}},$$

Where *d*_*il*_ is the distance between neuron *i* and the location of the stimulation contact *l* and *l*_*c*_ is the length of the electrode contacts. By setting *d*_min_ = 0.7 mm we avoid any singularity that could occur at *d*_*il*_ = 0. Hence, we prohibit possible neuron coordinates within a cylindrical volume with 1.4 mm diameter around the electrode axis.

**FIGURE 2:**
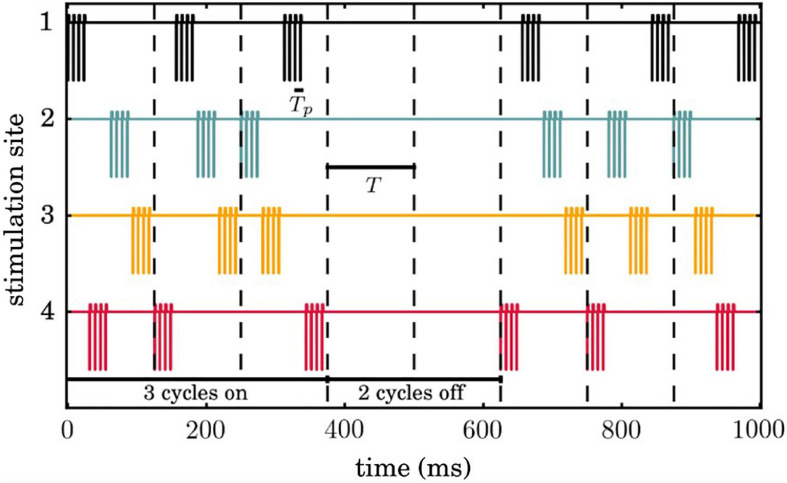
Temporal evolution of coordinated reset (CR) simulation signals. Successive 3 ON and 2 OFF cycles are indicated by vertical dashed lines and the temporal distance between two successive vertical lines correspond to the period *T*of each cycle. Each stimulation site is activated exactly once during an ON cycle, where the sequence of activation across stimulation sites is random.

We used *M* =  4 stimulation sites while all neurons receive input from all four sites. Figure 2 shows an example of our CR stimulation signal. During each stimulation period *T* = 125*ms*, each site is activated via an electric burst only once, and not at the exact same onset time with any other site. This order of activation varies randomly in every cycle (during the CR ON period). Three cycles of CR stimulation (ON-cycles) were followed by two cycles without stimulation (OFF-cycles) following Lysyansky et al. (2011) and Adamchic et al. (2014a). *The* stimulation period within a burst is set at *T*_p_ = 7.69ms, the CR stimulation amplitude κ = −3.3 is, the width of the cathodal pulse ω= 200μs and the duration and amplitude of the charge-balancing anodal pulse *p*_s_ = 8. The CR stimulation signal delivered at neuron *i* from stimulation contact *l* is given by the following equation:

(22) $$I_{\text{stim}}\left(t\right)=\sum_{l=1}^{M}S\left(d_{il}\right)\rho_{l}\left(t\right)P\left(t\right),$$

ρ_*l*_(*t*) is an indicator function representing the spatiotemporal activation of stimulation sites (set at 1 when the site is activated and 0 otherwise). For more details on the CR stimulation setup see Ebert et al. (2014).

### Macroscopic Measurement

In order to measure the degree of neuronal synchronization within the STN and GPe ensembles we use the order parameter (Kuramoto, 1984; Daido, 1992; Tass, 1999):

(23) $$R\left(t\right)=\left|N^{-1}\sum_{j}{\text{e}}^{i\varphi_{j}\left(t\right)}\right|,$$

Where *i* here denotes the unit imaginary unit $\sqrt{-1}$, φ_*j*_(*t*) =  2π(*t*−*t*_*j, m*_)/(*t*_*j, m* + 1_−*t*_*j, m*_)for *t*_*j, m*_≤*t* < *t*_*j, m* + 1_ is a linear approximation of the phase of neuron *j* between its *m*^*th*^ and (*m* + 1)^*th*^ spikes at spiking times *t*_*j,m*_ and *t*_*j,m+1*_. *R*(*t*) is influenced by the synaptic weights, as the latter are time dependent due to the STDP. The order parameter *R* measures the extent of phase synchronization in the neuronal ensemble and takes values between 0 (absence of in-phase synchronization) and 1 (perfect in-phase synchronization).

## Results

### Simulation Description and Protocols

We present the general simulation setups and protocol designs we employed throughout this study.

**FIGURE 3:**
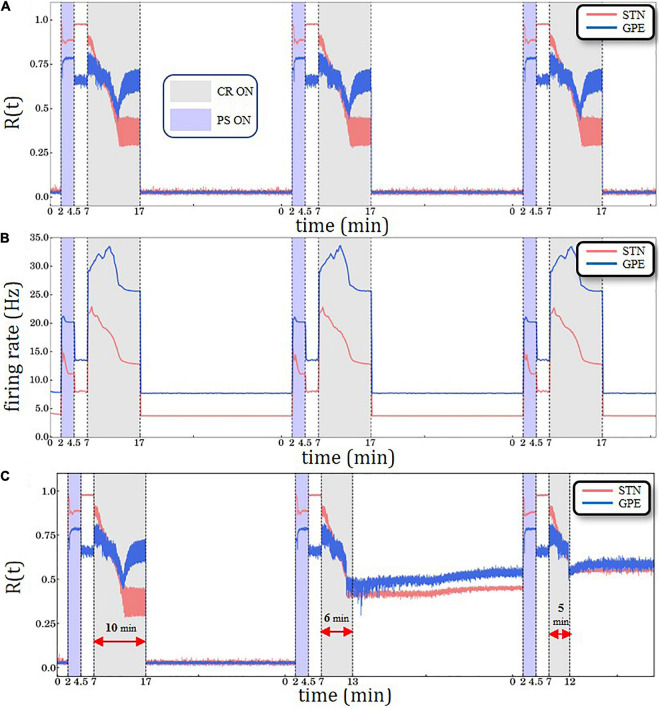
Periodic Stimulation (PS)-CR sequences without structural plasticity. **(A)** Time evolution of the order parameter *R*(*t*)averaged over a sliding window (10 ms) for STN (red solid line) and GPe (blue solid line) neurons. **(B)** Spiking rates for STN and GPe neurons for three identical CR periods. Light blue bands denote the PS (2.5 min) intervals and light gray the CR intervals (10 min). STDP is active throughout the entire trial. The synchronization-desynchronization process caused by the first PS-CR sequence is repeated by the two subsequent PS-CR sequences, indicating its reproducibility in the absence of PS. **(C)** PS-CR sequence with one long CR epoch followed by two PS-CR sequences with insufficient CR duration (6 and 5 min, respectively) without structural plasticity.

**FIGURE 4:**
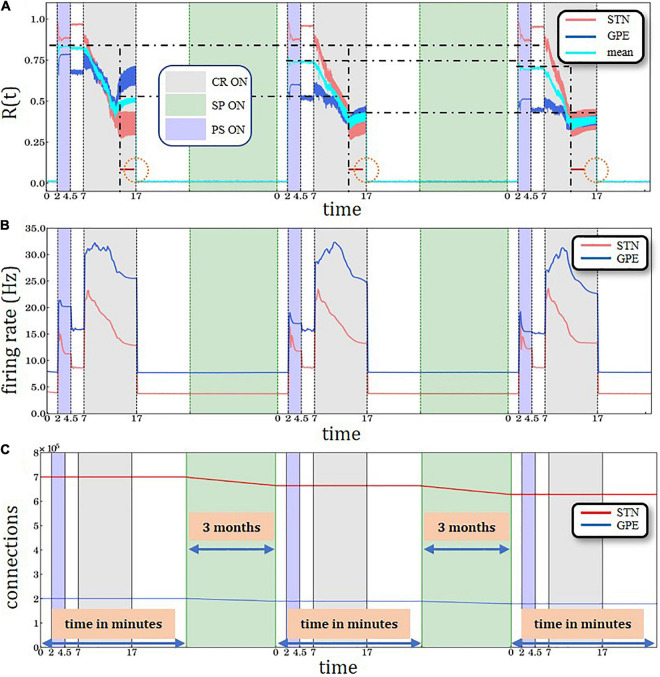
Three identical PS-CR sequences, with structural plasticity with 3.0 Hz target firing rate. **(A)** A sliding window of 100 simulation time steps at each different epoch when STDP or structural plasticity was active for STN (red solid line) and GPe (blue solid line) neurons and the average value for the whole network (cyan solid line). The horizontal back dot-dashed lines indicate the plateaus’ gradual decrease of the level of synchronization degree after each SP period interval. The vertical black dot-dashed depict the gradual improvement of the CR’ performance after each consecutive stimulation and SP cycle. The lines’ respective location is set at the beginning of the plateaus immediately after the initial sharp decrease. The equally sized horizontal red lines and the circle are used to provide a visual aid to this effect. **(B)** Spiking rates for STN and GPe neurons, respectively. PS, CR, STDP, and structural plasticity activation interval period. The color coding and time units are defined in the legends of panels **(A,C)**. **(C)** Time course of the total number of synaptic excitatory connections in the STN population.

**FIGURE 5:**
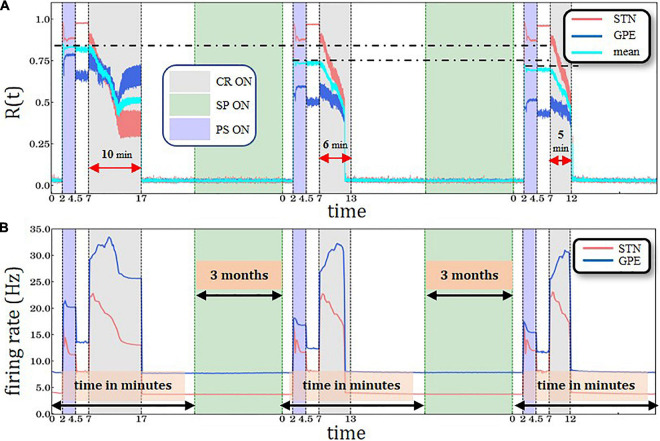
Periodic Stimulation-Coordinated Reset sequence with long CR epoch followed by two PS-CR sequences with reduced CR duration, with structural plasticity (SP) with 3.0 Hz target firing rate. **(A)** A sliding window of 100 simulation time steps at each different epoch when STDP or structural plasticity was active for STN (red solid line) and GPe (blue solid line) neurons and the average value for the whole network (cyan solid line). **(B)** Spiking rates for STN and GPe neurons, respectively. PS, CR, STDP, and structural plasticity activation interval period color coding and units are defined in Figure 3.

• We start in a desynchronized state with sufficiently weak synapses and large numbers of possible synaptic connections within the STN population (i.e., large number of dendritic spines) and corresponding firing rate of ∼4 Hz.
• To generate a synchronized reference state, we deliver sufficiently strong kindling-like PS to increase synaptic weights, so that the network is shifted to a strongly synaptically connected and strongly synchronized stable state. In several model networks kindling is achieved more rapidly than anti-kindling (Tass and Majtanik, 2006). The kindling process is achieved by PS of comparably short duration (*T*_PS_ =  2.5min).
• The PS-induced synchronized state is stable, so that both STN and GPe remain in a stable synchronized state after cessation of PS.
• CR stimulation is then delivered to the network in the synchronized state, which causes a desynchronization and a reduction of its synchrony (as measured by the order parameter).
• If CR stimulation is delivered for a sufficiently long time, during the CR-off period the network relaxes to a desynchronized state with down-regulated synaptic weights but still a large number of possible synaptic connections (i.e., large number of dendritic spines).
• Up to this point STDP is active for STN neurons.
• From this point on, two options are being explored. The system may either evolve only with STDP activated (Figure 3 and Supplementary Figure 2) or, alternatively, with structural plasticity activated between CR intervals (Figures 4, 5 and Supplementary Figures 1, 3, 4). In the latter case, we consider different scenarios that evolve depending on whether the target firing rate of the structural plasticity mechanism is modified by CR stimulation.

This elementary stimulation protocol comprises a PS-induced transition from a desynchronized to a synchronized state and a CR-induced reverse transition. In this PS-CR sequence, PS serves as a test stimulation assessing the network’s susceptibility to synchronizing stimulation. We use different combinations and variations of this elementary stimulation protocol. First, to demonstrate reversibility, we simply repeat the same PS-CR sequence again using the same CR-ON time period and the exact same number of connections. Second, after the first PS-CR sequence, we turn on structural plasticity in the desynchronized state for a computational time interval corresponding to arbitrary time units. In our implementation, STDP and structural plasticity operate at different time scales simulating biological time which ranges from milliseconds up to minutes when STDP is ON and up to several days when structural plasticity is ON. It is important to note that due to software constraints only one of the two plasticity mechanisms is allowed to be ON in a given time interval. Otherwise, the growth rate in the structural plasticity model could not be used to change the speed at which structural plasticity effects occur, which would render the model computationally intractable. In this way, the simulation does not mix the different physiological components and effects, taking place on different time scales. The structural plasticity model efficiently simulates relatively large biological time intervals in feasible computing times. Furthermore, it allows the system to evolve smoothly enough, maintaining its dynamical stability during the synapse rewiring process. The simulated structural plasticity period of approximately 20 min computing time corresponds to a biological time that ranges from days to weeks (Butz et al., 2014). This enables a slow dynamical evolution of the network’s topology, governed by the structural plasticity homeostatic rule with pre-defined or stimulation-induced target parameters related to non-pathological neuronal spiking rates. The subsequent second PS-CR sequence acts like a probe, detecting PS-induced alterations of the network’s synchronizability and desynchronizability.

In this study, we configured the structural plasticity algorithm as follows: we use Gaussian growth curves and set ν_SP_ = 0.00008 synaptic elements/ms for the STN-STN connections, ν_SP_ = 0.00002 synaptic elements/ms for the STN-GPe connections and ε = 3.0Hz for both pre-synaptic elements. We note that the units of the growth rate ν_*SP*_ are defined here in synaptic elements/ms of simulation time. Biologically realistic values of the growth rate ν_*SP*_ would be at least two orders of magnitude slower than the ones used in this manuscript. The use of these higher values allows us to have faster changes in the connectivity while keeping the normal simulation step of NEST and reduce the simulation time representing the structural changes which would be observed in extended biological times. This setup is meant to drive the target STN firing rate toward a non-pathological rate of about 3.0 Hz. In our implementation, structural plasticity is applied only to excitatory connections originating from the STN population (see “Materials and Methods” section for a detailed description). The growth rate has positive values because the synapses are excitatory. The curve specifies that when neurons have a firing rate higher than 3.0 Hz, synapses will be deleted; if neurons have a firing rate lower than 3.0 Hz, synapses will be created. Rewiring occurs when a synaptic element is deleted together with its corresponding synapse, but the matching synaptic element in the other neuron remains available for a new connection with a new matching element. We provide a rewiring factor in the form of Gaussian curves for the post-synaptic elements of both connections of ν_SP_ = −0.005 synaptic elements/ms. This relatively higher value of ν_*SP*_ compared to the ones in the pre-synaptic growth curves allows the formation of several potential new contact points to which deleted connections can be rewired. In our study, we observed a firing rate of approximately 3.6 Hz at the moment structural plasticity is enabled after each PS-CR sequence. Note, the firing rate is the STN network’s actual firing rate which is measured by the structural plasticity algorithm and taken into account for the generation/deletion of synaptic elements, based on the structural plasticity homeostatic rule. The chosen configuration assumes that as a result of a CR epoch the target firing rate of the STN population is reset to 3.0 Hz, which reflects a “healthy” firing rate. This means that during the simulation, synapses will slowly be deleted until the desired 3.0 Hz target is reached. We have experimented with a broad range of parameter combinations before choosing these values. The results discussed here use a configuration within a range of parameters which show memory effects, see Supplementary Material for more details. The major rationale was to design a structural plasticity mechanism whose time evolution causes sufficient synaptic deletion in order to impact on the network’s macroscopic dynamics (as measured by the order parameter) within computationally feasible times.

As already mentioned, the structural plasticity algorithm measures and takes into account the current STN firing rate and modifies the synaptic connectivity until the target (3.0 Hz) is reached. With the selected parameters, on each period of simulation with structural plasticity approximately 5.33% of the total STN-STN connections and 4.8% of the total STN-GPe connections are effectively deleted. We then apply a second, identical PS-CR sequence or a modified PS-CR sequence. To this end, we either keep the same CR-ON period or gradually shorten it to 6 and 5 min. After a sufficient amount of time STDP does no longer change the order parameter and mean firing rate. Based on a series of simulations it turned out that a 10 min post-CR window with STDP ON is sufficient for the network to attain a stable state as characterized by stable order parameter and firing rate. We then turn on structural plasticity and turn off STDP, since an increased growth rate is required to speed structural plasticity simulations up to achieve feasible computation times.

### Repeated Delivery of PS-CR Sequences With STPD Only

Anti-kindling requires CR stimulation to be delivered at sufficient amplitude and duration (Tass and Majtanik, 2006; Hauptmann and Tass, 2009; Popovych and Tass, 2012; Ebert et al., 2014). Figure 3A shows the overall desynchronization effect for sufficiently strong CR stimulation amplitude κ=−3.3 (see Ebert et al., 2014 for details). We synchronize and desynchronize the network repetitively by delivering three PS-CR sequences (see “Results” Section: Simulation Description and Protocols). The network starts in a desynchronized state, and then gets synchronized by PS. PS epochs are highlighted by light blue color bands, ranging from $t_{\text{start}}^{PS}=2$ min to $t_{\text{stop}}^{PS}=4.5$min. After PS delivery the network first evolves spontaneously; that is, without further stimulation. Next, the PS-CR sequence is completed by administration of CR stimulation. CR epochs are indicated by light gray color bands, ranging from $t_{\text{start}}^{CR}=7$ min to $t_{\text{stop}}^{CR}=17$min. A 10 min CR epoch is considered a “long” CR period. Subsequently, the network again evolves spontaneously, thereby remaining in a desynchronized state. Afterward, two more PS-CR sequences are delivered. Note, at the beginning of each PS-CR sequence, we reset the time back to zero, while the simulation proceeds in a continuous manner. Resetting the time scale is performed in order to facilitate successive comparisons with altered plasticity mechanisms and/or CR stimulation durations. In Figure 3B, we show the corresponding time evolution of the spiking rates of the STN (red curve) and GPe (blue curve) neurons, starting at ∼4 Hz/∼8 Hz (healthy model state), increasing to ∼15 Hz/∼21 Hz (pathological model state) and up to ∼23 Hz/∼34 Hz (during the CR epoch) and decreasing back to ∼4 Hz/∼8 Hz (after the PS-CR sequence, in the healthy model state). This process is repeated for each of the three PS-CR sequences in a reproducible manner, based on the macroscopic quantities used for assessment of overall synchrony and mean spiking rate. In the Supporting Information Section, we present a similar simulation with three PS-CR sequences with insufficiently strong CR stimulation amplitude (κ = −2.0) (Supplementary Figure 2); despite appropriate stimulation duration, the administered CR stimulation is not sufficient to desynchronize the STN-GPe circuit. The transient behavior of all macrovariables, STN *R*(*t*), GPe *R*(*t*) as well as STN and GPe mean firing rates, during the CR epoch depends on model parameters, e.g., on the plasticity ratio between depression and potentiationγ -value and CR stimulation intensity κ -value. For instance, for smaller γ -values (e.g., γ≲1.3 and fixed κ = −3.3) and identical CR stimulation duration there is no re-increase of the order parameter of GPe.

In general, in stimulated neural networks with STDP the values of macrovariables, such as order parameter *R*(*t*), mean synaptic weight, mean firing rate, may substantially depend on whether stimulation is ON and specifics of stimulus parameters and protocols (Manos et al., 2018a,b). In the PS epoch vs. the pause between the PS and the CR epoch the system approaches stable states that differ with respect to their order parameters and mean firing rates. In the model under consideration, depending on model and stimulus parameters different transients may occur. We do not have a theory or mechanistic explanation for the transitory decrease and re-increase of GPe *R*(*t*) observed for the parameters under consideration.

Next, we consider the impact of insufficiently short CR epochs (Figure 3C). To this end, the first PS-CR sequence with sufficient CR duration (10 min) is followed by two PS-CR sequences with insufficient CR duration (5 and 6 min, respectively). The short CR epoch does not cause a full-blown desynchronization. Rather, it shifts the network to a stable state with somewhat reduced synchronization. The effects of the second PS-CR sequence are reproduced by the third PS-CR sequence. This illustrates that the STN-GPe network is multistable. Apart from a fully synchronized state and a fully desynchronized state, we observe stable states with intermediate levels of synchronization. The short CR epochs only shift the network to such an intermediate state, not to a fully desynchronized state.

### Impact of Structural Plasticity on Three PS-CR Sequences

To study whether structural plasticity effects may accumulate despite interspersed PS-CR sequences, we administered three identical PS-CR sequences with long CR epochs and keep the target firing rate at 3.0 Hz (Figures 4A,B). Here, we also present the average (combined STN and GPe populations) order parameter value for the whole network is shown by the cyan solid line. The horizontal black dot-dashed lines indicate the plateaus’ gradual decrease of the level of synchronization degree after each structural plasticity period interval. In addition, lower levels of the order parameters and mean firing rates are reached faster during the third CR epoch compared to the second CR epoch, indicating cumulative structural plasticity effects. The vertical black dot-dashed depict the gradual improvement of the CR’ performance after each consecutive stimulation and SP cycle. The lines’ respective location is set at the beginning of the plateaus immediately after the initial sharp decrease. The equally sized horizontal red lines and the circle are used to provide a visual aid to this effect. The time course of the total number of synaptic excitatory connections in the STN population is shown in Figure 4C.

The cumulative effect of structural plasticity is further illustrated by delivering one PS-CR sequence with long CR epoch (10 min) followed by two PS-CR sequences with shorter CR duration (5 and 6 min) (Figure 5). We observe the same stepwise decrease of the post-PS STN synchronization (Figure 4A) and firing rates (Figure 5B). During the third CR epoch STN and GPe synchronization reaches lower levels and approaches steady state levels more rapidly compared to during the second CR epoch (Figure 5A).

Several model parameters are drawn from Gaussian distributions (see Supplementary Material). To check whether the model’s dynamics is robust with respect to variations of these parameters, for all relevant findings we have run several simulations, even if we presented only one. In fact, a relevant limitation of the structural plasticity model mechanism used in our manuscript is its computational cost which is even demanding for the JURECA supercomputer center at Jülich Research Center. To illustrate the robustness of our findings, we ran 20 random initializations for the simulation with structural plasticity and one PS-CR period (see Supplementary Figure 4).

Structural plasticity with sufficiently small target firing rate reduces the maximum values of STN synchronization. This is because the maximally achievable synchronization, as assessed by the order parameter, depends on the number of excitatory synapses. The more excitatory synapses can attain maximum connectivity values the greater the order parameter, see, e.g., Taylor (2012) and Townsend et al. (2020) and references therein. For illustration, let us consider the effect of a plain removal of a fraction of randomly selected STN-STN connections after initializing the network in a synchronized state (Supplementary Figure 5). To this end, we initialized our model in a synchronized state by delivering PS, with STDP turned ON and structural plasticity permanently turned OFF. We then turn off STDP, randomly deleted between 5 and 20% percentage of the excitatory STN-STN connections and evaluated the amount of synchronization in a 2 min time window. With increasing percentage of deleted STN-STN connections the amount of STN synchronization continuously decreases (Supplementary Figure 5).

## Discussion

This is the first computational study investigating the impact of structural plasticity on the outcome of CR stimulation. We implemented structural plasticity in a STN-GPe network with plastic STN-STN synapses governed by STDP. In the absence of structural plasticity, repeated administration of PS-CR sequences leads to reversible transitions between desynchronized and synchronized states (Figures 3A,B). By the same token, delivering PS-CR sequences with CR epochs of insufficient duration causes transitions between synchronized states of different amount of synchrony (Figure 3C and Supplementary Figure 2). To date, changes of susceptibility to CR stimulation after stimulation-free pauses have not been tested in computational studies. However, such memory-like phenomena were previously observed in a clinical study with vibrotactile CR stimulation (Pfeifer et al., 2021) and acoustic CR stimulation (Tass et al., 2012a). In general, effects of this kind may allow the further reduction of the total stimulation duration when designing therapeutic treatment regimes. In an example such as DBS, this could further reduce the occurrence or severity of AE in treated patients, which could significantly improve their quality of life. It is not straightforward to relate our measures, such as the order parameter, to clinical outcome scores. However, it was shown that CR-DBS-induced changes of UPDRS motor score (normalized to baseline) were significantly correlated with changes of peak beta power of the local field potential (LFP) (normalized to baseline) measured by the very STN contacts used for stimulation (Adamchic et al., 2014a). This correlation was obtained off medication, i.e., after proper withdrawal of Parkinson’s medication. Furthermore, Pfeifer et al. (2021) revealed that after 3 months of vCR therapy the off medication high beta band power in the sensorimotor cortex decreased significantly. Hence, after 3 months of vCR the sensorimotor cortex’ ability to resynchronize after profound medication withdrawal was limited. The 3-month interval corresponds to clinical findings obtained in a study with pre-defined timing of clinical visits (Pfeifer et al., 2021) and, hence, can only provide a first time scale approximation. The approximative 3-month time scale is also in agreement with findings obtained in a clinical proof of concept study with acoustic CR stimulation for the treatment of tinnitus (Tass et al., 2012a).

### Main Findings and Conclusion

The goal of our study was to demonstrate that clinically observed phenomena can be reproduced by incorporating structural plasticity as opposed to taking into account STDP alone. Our model qualitatively reproduces previously observed memory-like phenomena (Tass et al., 2012a; Pfeifer et al., 2021) if we assume that CR stimulation epochs cause a reduction of the target firing rate. Comparable memory effects of previous treatment sessions come into play when structural plasticity is activated with appropriate target firing rates (Figures 4, 5). On one hand, the PS epochs in the PS-CR sequences may serve as a standardized model process accounting for detrimental influences on patients during stimulation-free intervals, leading to a re-increase of symptoms (see Tass et al., 2012a). On the other hand, the PS epochs may be considered as standardized probes, enabling the testing of the network’s resistance to synchronizing stimulation protocols. Furthermore, we showed that simulation setups which account only for STDP (Figure 3) are not able to exhibit this type of cumulative effect observed in clinical trials, acting on time scales of months (see, e.g., Figure 8 in Pfeifer et al., 2021).

In this study we have taken into account STDP and structural plasticity by two separate mechanisms. STDP changes synaptic weights according to the timing relationship of the corresponding pre- and postsynaptic neurons on a fast time scale (Gerstner et al., 1996; Markram et al., 1997; Bi and Poo, 1998, 2001; Song et al., 2000; Ebert et al., 2014), whereas structural plasticity adapts the single neuron’s firing rate to a target firing rate by creating and deleting synapses on a slow time scale (Butz and van Ooyen, 2013; Diaz-Pier et al., 2016), as discussed above. So far, in other modeling studies certain types of structural plasticity mechanisms and STDP were simultaneously implemented at a more theoretical level, making connections to Hamiltonian sampling (Yu et al., 2016) or Bayesian inference mechanisms (Kappel et al., 2015). In contrast, in our study, we separated the two mechanisms for computational convenience, efficiency and performance, since in a first approximation we assume that no new synapses will be created or pre-existing synapses will be deleted during the short long CR epochs. In contrast, we simulate the slow structural plasticity effects initiated by the brief CR epochs. However, in future studies firing pattern-dependent mechanisms such as STDP may also be included in the structural plasticity model in order to account for selective, firing pattern-guided creation or deletion of synapses (Clopath et al., 2010; Legenstein and Maass, 2011; Perin et al., 2011; Zheng et al., 2013). The actual structural plasticity model will certainly impact on the design of optimal stimulation patterns which cause long-lasting desynchronization by therapeutic synaptic rewiring, achieved by adequately controlling collective firing patterns as well as firing rates. Note, in our model we incorporated plasticity mechanisms, STDP and structural plasticity, in the directly stimulated target area, the STN, only.

The numerically implemented SP mechanism in our simulations provides a plausible explanation of the physiological memory-like effects. However, this approach requires an appropriate parameter tuning in order to demonstrate such effects within computationally reasonable time scales. Assuming that the target firing rate is sufficiently low (3.0 Hz), characterizing a healthy STN firing state, structural plasticity continuously down-regulates the firing rate by deleting excitatory synapses. Consequently, during the PS epoch the STN re-synchronization is less pronounced and does not reach the initial levels observed by the first PS epoch (Figure 4A). The level of synchronization of the GPe network, which provides an inhibitory output, during and directly after the PS epoch, does not change substantially compared to the first PS epoch (Figure 4A). However, during the subsequent CR epoch the desynchronization of STN and GPe are both accelerated in a similar manner compared to the first CR epoch (Figure 4A). Accordingly, a long-lasting desynchronization of STN and GPe can be achieved by means of a considerably shorter CR epoch (Figure 4A). An important prerequisite of the structural plasticity effect is that after the CR epoch the STN firing rate relaxes to a value (3.6 Hz) sufficiently close to the structural plasticity’s target firing rate (3.0 Hz). This enables structural plasticity to take over efficiently and down-regulate the number of excitatory connections within the STN, together with the firing rate using the homeostatic rule. The choice of a Gaussian curve in the structural plasticity model employed in this study (Bos et al., 2015; Diaz-Pier et al., 2016) was motivated by experimental data (Lipton and Kater, 1989; Al-Mohanna et al., 1992) which suggests that the generation of new spines starts slowly when the activity in the neuron is low and progressively increases to a maximum. Then it starts to decay until a homeostatic equilibrium is reached (Butz and van Ooyen, 2013). During development the degree of plasticity during critical periods also increases slowly until a maximum level, and then decreases until a degree of maturity is achieved (see box 1 in Hensch, 2005). Note, a reduction of approximately 5.76% of STN-STN connections together with the rewiring of connections during the structural plasticity epoch are sufficient to increase the network’s resistance to PS and also its increased susceptibility to CR stimulation; thus, the structural plasticity effects accumulate, even though intersected by a PS-CR sequence (Figure 5).

In this study with our novel NEST based code, we combine the power of structural and synaptic plasticity providing a modeling test environment that enables us to capture memory-like phenomena of susceptibility to CR stimulation. From the modeling and simulation perspectives, there are several challenges related to this setup; the most significant is that simulations of detailed networks are computationally demanding, especially when the two types of plasticity are involved. This is mainly due to the variations in the transmission and generation of spiking events in the network, as well as the additional calculations that take place at the synapse level. Simulations of the size that we report in this study can have a simulation to real time factor of about 10–20. This means that simulations of 1 s take 10–20 s to be simulated computationally. Accordingly, exploring slow structural plasticity induced changes of a simulated network within 1 month of biological time, could take a normal simulation of up to several (∼20) months duration. Obviously, such a scenario is significantly resource-intensive. In contrast, using our NEST simulations, we can accelerate the effects of structural plasticity so that simulations can be reduced from months to a couple of hours. With this solution, we are able to simulate at feasible computing times both the synaptic plasticity, which takes place during PS and CR epochs, followed by a stabilization period and the subsequent activation of structural plasticity, running at reasonably high speed by means of an accelerated model. During this accelerated simulation period, we disable synaptic plasticity in order to avoid inconsistencies that would be induced due to the fact that the model operates at different time scales. By combining both types of plasticity in such a temporally interleaved way, we are able to explore the immediate stimulation-induced effects of synaptic plasticity as well as the long-term effects mediated by structural plasticity within a single consistent, efficient, and tractable simulation model.

Future studies could be designed to investigate the development of desynchronizing stimulation protocols that are adapted to specific ranges of target firing rates. It is therefore crucial to understand stimulation-induced changes of the firing rate. In fact, with very few exceptions (see, e.g., Lysyansky et al., 2011), the vast majority of computational studies devoted to CR stimulation to date have not focused on stimulation-induced changes of the firing rate. Our study highlights that not only the CR epochs may be crucial for the long-term stimulation outcome; to the best of our knowledge, our study is the first to also demonstrate that pauses in between effective CR epochs also play an active and critical role. CR stimulation and, in general, all kinds of efficient desynchronizing stimulation protocols may initiate STN-GPe circuits in favorable states, so that structural plasticity makes networks more susceptible to desynchronizing stimulation over time. In a previous computational study in a neural network with STDP, but no structural plasticity, it was shown that spaced CR stimulation (i.e., CR stimulation intermingled with sufficiently long stimulation-free pauses) may significantly improve efficacy; this is particularly important if CR stimulation is delivered at otherwise ineffectively weak intensities (Popovych et al., 2015). Together with our current study, this illustrates the importance and active role of pauses in therapeutic processes employing plasticity principles. Stimulus-free intervals should not just be considered periods without intervention. Rather they may be an integral part of a therapeutic process enabling and potentiating stimulus effects which should be adequately addressed by dose-finding studies. In future studies we plan to incorporate plasticity mechanisms in additional brain areas. However, given the huge amount of computation time required to networks with STDP and structural plasticity, one might modify and further adapt structural plasticity model mechanisms to given high-performance computing architectures.

In summary, in our proof of concept-like computational study we added structural plasticity to an existing STN-GPe model which, so far, only comprised STDP in the STN (Ebert et al., 2014). The STN is an excitatory population (Shink et al., 1996). To model STDP for STN’s efferent excitatory synapses, we have used a standard STDP model which has been used in numerous other studies (see, e.g., Gerstner et al., 1996; Gerstner and Kistler, 2002; Dan and Poo, 2004; Sjöström et al., 2008). In future studies both structural plasticity and STDP should also be taken into account for the GPe. Unlike STN, GPe is an inhibitory population (Shink et al., 1996). The structural plasticity mechanism used in this manuscript (Butz and van Ooyen, 2013; Butz et al., 2014) can also be applied to inhibitory synapses. However, to model STDP in inhibitory synapses, appropriate rules of rules of inhibitory STDP have to be taken into account (Vogels et al., 2013).

## Code and Scripts Availability

The NEST version used for the simulations presented in this manuscript can be found here: https://github.com/sdiazpier/nest-simulator.git (branch dbs_sp) and the model scripts can be found in this repository: https://github.com/sdiazpier/dbs_sp.git.

## Data Availability Statement

The original contributions presented in the study are included in the article/Supplementary Material, further inquiries can be directed to the corresponding authors.

## Author Contributions

TM, SD-P, and PT conceived and designed the experiments, analyzed the data, and wrote the manuscript. TM and SD-P performed the experiments and contributed reagents, materials, and analysis tools. All authors contributed to the article and approved the submitted version.

## Conflict of Interest

PT works as consultant for Boston Scientific Neuromodulation and Gretap AG and is inventor or co-inventor on a number of patents for invasive and non-invasive neuromodulation. The remaining authors declare that the research was conducted in the absence of any commercial or financial relationships that could be construed as a potential conflict of interest.

## Publisher’s Note

All claims expressed in this article are solely those of the authors and do not necessarily represent those of their affiliated organizations, or those of the publisher, the editors and the reviewers. Any product that may be evaluated in this article, or claim that may be made by its manufacturer, is not guaranteed or endorsed by the publisher.
