# Supplementary material for: Long-Term Desynchronization by Coordinated Reset Stimulation in a Neural Network Model With Synaptic and Structural Plasticity
Source: Front Physiol. 2021 Sep 8;12:716556. doi: 10.3389/fphys.2021.716556 (PMC8455881; doi:10.3389/fphys.2021.716556)
Supplement: Supplementary file 1 [file Data_Sheet_1.docx]

Supplementary Material

**Supplementary Table 1.** Parameter values used for STN neurons. The maximum conductances $g_{\mathrm{ion}}$and reversal potentials $v_{\mathrm{ion}}$were Gaussian distributed with a standard deviation of 5$\%$ around the respective mean values given is this table.

| $g_{L}$ | 2.25 nS/*μ*m^2^ | $v_{L}$ | -60.0 mV |
| --- | --- | --- | --- |
| $g_{K}$ | 45.0 nS/*μ*m^2^ | $v_{K}$ | -80.0 mV |
| $g_{\mathrm{Na}}$ | 37.5 nS/*μ*m^2^ | $v_{\mathrm{Na}}$ | 55.0 mV |
| $g_{\mathrm{Ca}}$ | 0.5 nS/*μ*m^2^ | $v_{\mathrm{Ca}}$ | 140.0 mV |
| $g_{\mathrm{ahp}}$ | 9.0 nS/*μ*m^2^ | $v_{\mathrm{ss}}$ | 0.0 mV |
| $g_{T}$ | 0.5 nS/*μ*m^2^ | $v_{\mathrm{sg}}$ | 0.0 mV |
| $\tau_{h}^{1}$ | 500.0 ms | $\tau_{h}^{0}$ | 1.0 ms |
| $\tau_{n}^{1}$ | 100.0 ms | $\tau_{n}^{0}$ | 1.0 ms |
| $\tau_{r}^{1}$ | 17.5 ms | $\tau_{r}^{0}$ | 40.0 ms |
| $\varphi_{h}$ | 0.75 | $\varphi_{n}$ | 0.75 |
| $\varphi_{r}$ | 0.2 |  |  |
| $\tau_{ss}$ | 1.0 ms | $\tau_{gs}$ | 3.3 ms |
| $\theta_{m}$ | -30.0 | $o_{m}$ | 15.0 |
| $\theta_{h}$ | -39.0 | $o_{h}$ | -3.1 |
| $\theta_{n}$ | -32.0 | $o_{n}$ | 8.0 |
| $\theta_{r}$ | -67.0 | $o_{r}$ | -2.0 |
| $\theta_{a}$ | -63.0 | $o_{a}$ | 7.8 |
| $\theta_{b}$ | 0.4 | $o_{b}$ | -0.1 |
| $\theta_{s}$ | -39.0 | $o_{s}$ | 8.0 |
| $\theta_{h}^{\tau}$ | -57.0 | $o_{h}^{\tau}$ | -3.0 |
| $\theta_{n}^{\tau}$ | -80.0 | $o_{n}^{\tau}$ | -26.0 |
| $\theta_{r}^{\tau}$ | 68.0 | $o_{r}^{\tau}$ | -2.2 |
| $k_{1}$ | 15.0 | $k_{\mathrm{Ca}}$ | 22.5 |
| $\Gamma$ | 3.75 $\times$10^-5^ms^-1^ | $c_{m}$ | 1 pF/μm^2^ |

**Supplementary Table 2.** Parameter values used for GPe neurons. The maximum conductances $g_{\mathrm{ion}}$and reversal potentials $v_{\mathrm{ion}}$were Gaussian distributed with a standard deviation of 5$\%$ around the respective mean values given is this table.

| $g_{L}$ | 0.1 nS/*μ*m^2^ | $v_{L}$ | -55.0 mV |
| --- | --- | --- | --- |
| $g_{K}$ | 30.0 nS/*μ*m^2^ | $v_{K}$ | -80.0 mV |
| $g_{\mathrm{Na}}$ | 120.0 nS/*μ*m^2^ | $v_{\mathrm{Na}}$ | 55.0 mV |
| $g_{\mathrm{Ca}}$ | 0.15 nS/*μ*m^2^ | $v_{\mathrm{Ca}}$ | 120.0 mV |
| $g_{\mathrm{ahp}}$ | 30.0 nS/*μ*m^2^ | $v_{\mathrm{gg}}$ | -80.0 mV |
| $g_{T}$ | 0.5 nS/*μ*m^2^ | $v_{\mathrm{gs}}$ | -100.0 mV |
| $\tau_{h}^{1}$ | 0.27 ms | $\tau_{h}^{0}$ | 0.05 ms |
| $\tau_{n}^{1}$ | 0.27 ms | $\tau_{n}^{0}$ | 0.1 ms |
| $\tau_{r}$ | 30.0 ms |  |  |
| $\varphi_{h}$ | 0.05 | $\varphi_{n}$ | 0.05 |
| $\varphi_{r}$ | $1.0$ |  |  |
| $\tau_{\mathrm{gg}}$ | 3.3 ms | $\tau_{\mathrm{sg}}$ | 1.0 ms |
| $\theta_{m}$ | -37.0 | $o_{m}$ | 10.0 |
| $\theta_{h}$ | -58.0 | $o_{h}$ | -12.0 |
| $\theta_{n}$ | -50.0 | $o_{n}$ | 14.0 |
| $\theta_{r}$ | -70.0 | $o_{r}$ | -2.0 |
| $\theta_{a}$ | -57.0 | $o_{a}$ | 2.0 |
| $\theta_{s}$ | -35.0 | $o_{s}$ | 2.0 |
| $\theta_{h}^{\tau}$ | -40.0 | $o_{h}^{\tau}$ | -12.0 |
| $\theta_{n}^{\tau}$ | -40.0 | $o_{n}^{\tau}$ | -12.0 |
| $k_{1}$ | 30.0 | $k_{\mathrm{Ca}}$ | 20.0 |
| $\Gamma$ | 1 $\times$10^-4^ms^-1^ | $c_{m}$ | 1 pF/μm^2^ |

**Comparison of different system sizes**


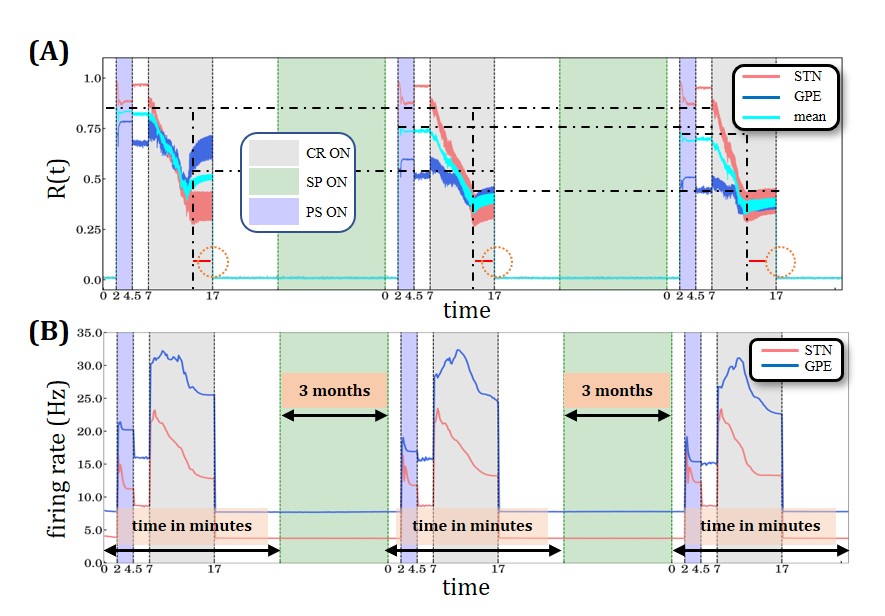


**Supplementary Figure 1. The stimulation protocol from Figure 3 was performed in the network with two system sizes and correspondingly scaled connectivity probabilities.** Original model (Ebert et al., 2014) with larger system size, containing 10,000 STN neurons and 10,000 GPe neurons with connection probability within and between populations of 7%. **(A)** a sliding window of 100 simulation time steps at each different epoch when STDP or structural plasticity was active for STN (red solid line) and GPe (blue solid line) neurons and the average value for the whole network (cyan solid line). The horizontal back dot-dashed lines indicate the plateaus’ gradual decrease of the level of synchronization degree after each SP period interval. The vertical black dot-dashed depict the gradual improvement of the CR’ performance after each consecutive stimulation and SP cycle. The lines’ respective location is set at the beginning of the plateaus immediately after the initial sharp decrease. The equally sized horizontal red lines and the circle are used to provide a visual aid to this effect. **(B)** Spiking rates for STN and GPe neurons, respectively. There is no qualitative difference of the stimulus response behavior of both models as assessed by macroscopic quantities, order parameter and mean firing rate. PS, CR, STDP and structural plasticity activation interval period color coding and units are defined in **Figure 4.**

**Inadequate CR stimulation amplitude**


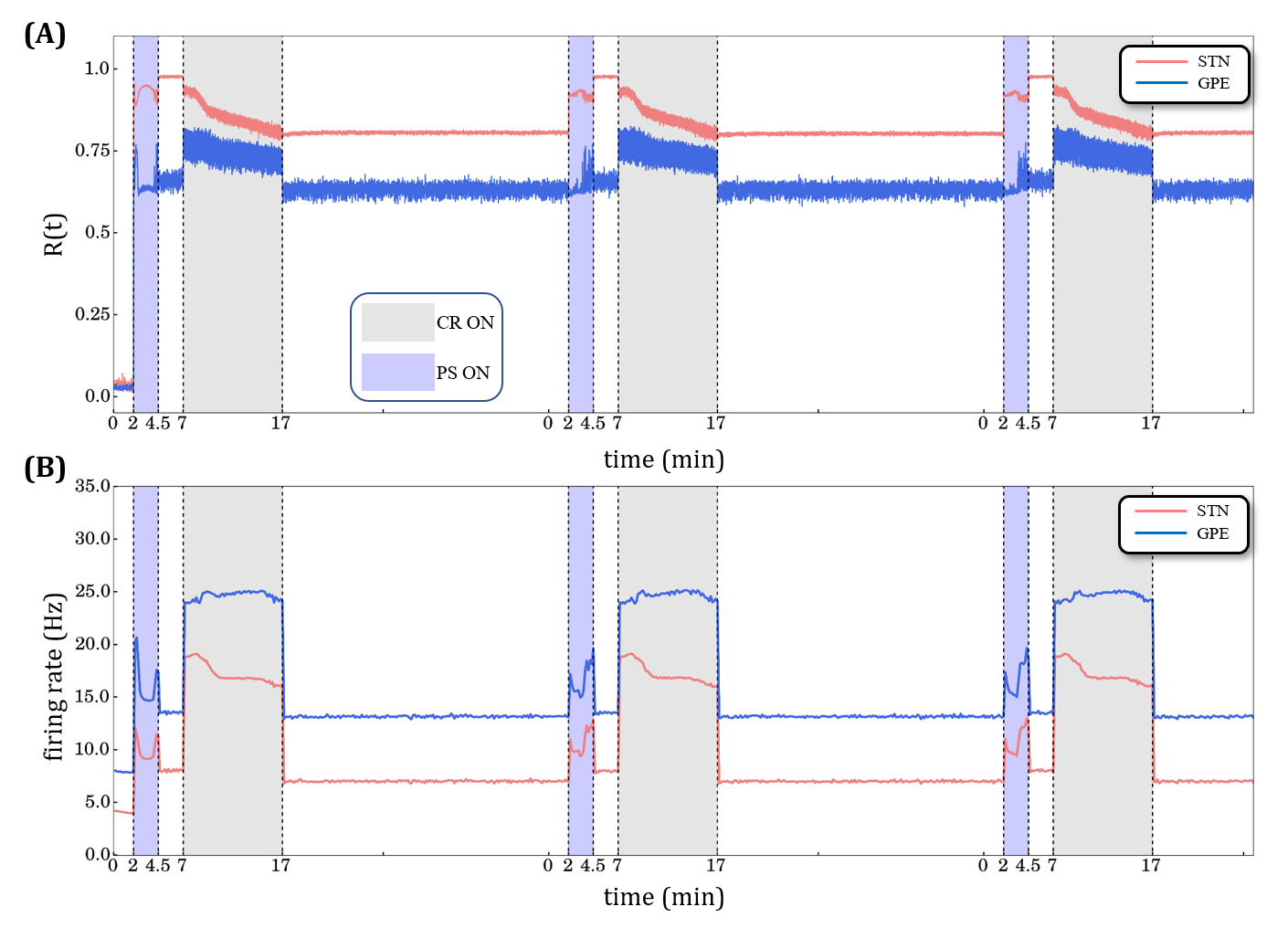


**Supplementary Figure 2.** **Desynchronization and spiking rate for 3 long CR sessions without structural plasticity with weak stimulation amplitude** $\boldsymbol{\kappa=-2.0}$**.** **(A)** Time evolution of the order parameter $R(t)$averaged over a sliding window (10 ms) for STN (red solid line) and GPe (blue solid line) neurons. **(B)** Spiking rates for STN and GPe neurons respectively. Light blue bands denote the Periodic Stimulation (2.5 min) intervals and light grey the CR-ON stimulation periods (10 min). STDP is active throughout the whole-time evolution.

**Structural plasticity and rewiring parameter (synapse creation & deletion)**


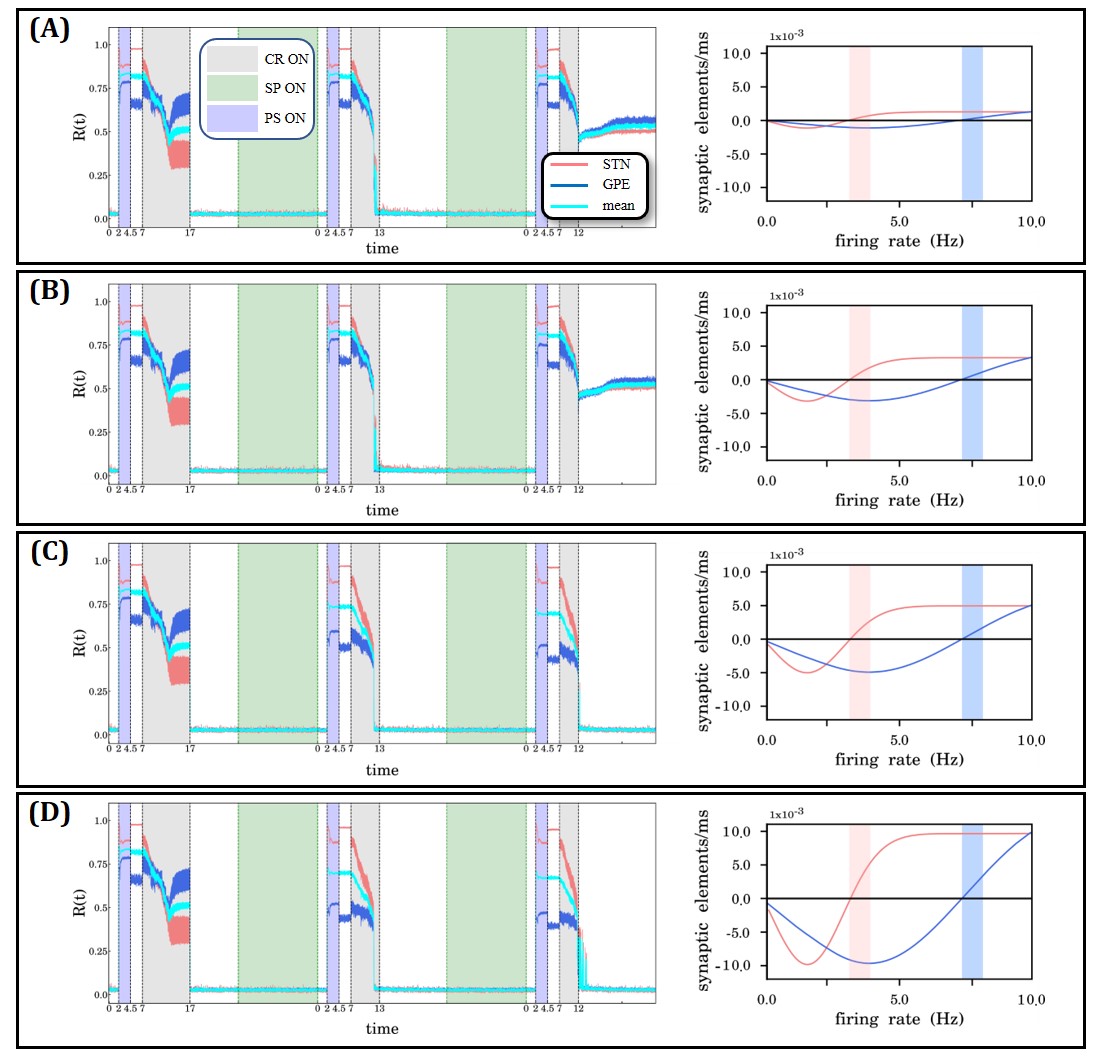


**Supplementary Figure 3.** **Parameter exploration on the rewiring factor of the structural plasticity rules.** The rewiring is implemented as a Gaussian growth curve at the post-synaptic element of the STN-STN (red) and STN-GPe (blue) connections. A rewiring factor is set at **(A)** $-0.001$, **(B)** $-0.003$, **(C)** $-0.005$, and **(D)** $-0.01$. The light red vertical bar and the light blue vertical indicate the activity operation intervals of the STN and GPe neurons respectively. The growth rate of the pre-synaptic elements for both STN-STN and STN-GPe connections was fixed to $0.00008$ and $0.00002$ respectively (same setup as shown in **Figure 1D** and in all other cases with SP in the manuscript). Panels (A) and (B) depict relatively weak memory effects. In panel (C) we present the rewiring parameter values that we use in the main body of the manuscript providing strong enough memory effect to achieve desynchronization in the short third CR stimulation period. Panel (D) also shows relatively strong memory effect but not substantially different than the ones in panel (C). Thus, we consider a rewiring factor with value set at $-0.005$ marks the beginning of a parameter value range within which the SP demonstrates relatively strong memory effects.

**Robustness of findings**


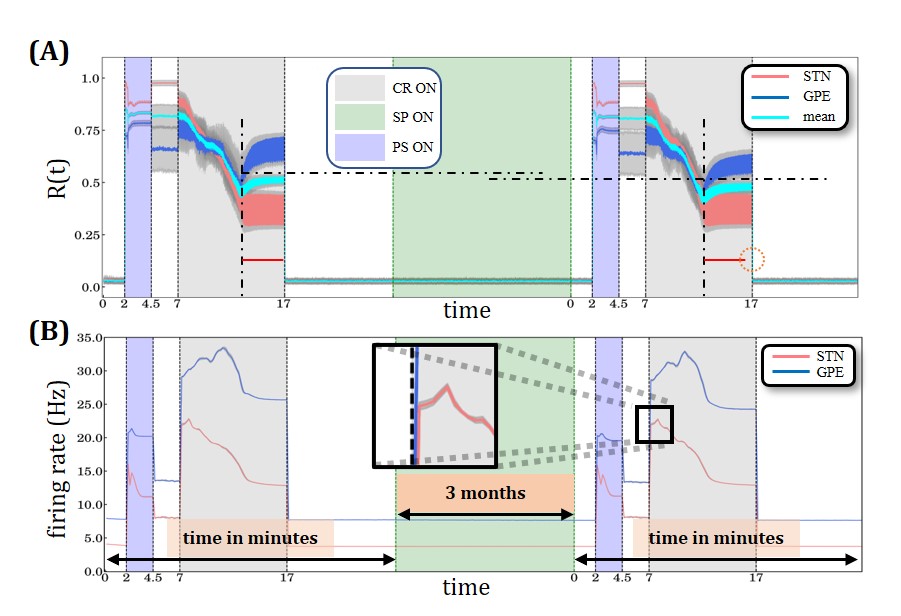


**Supplementary Figure 4. Two identical Periodic Stimulation (PS)-Coordinated Reset (CR) sequences, with structural plasticity. (A)** Time evolution of the order parameter averaged over a sliding window (1000 ms) for STN (red solid line) and GPe (blue solid line) for 20 initializations with different seeds for the random number generator with corresponding standard deviation (gray). **(B)** Spiking rates for STN and GPe neurons, respectively. PS, CR, STDP and structural plasticity activation interval period color coding and units are defined in **Figure 4.** The inset depicts the comparably small values of the standard deviation around the mean firing rate (more evident for the STN population). **(C)** Time course of the total number of synaptic excitatory connections in the STN population. Note, standard deviation of total number of excitatory connections across the 20 simulations amounts to only 0.057 % (i.e., 378 synapses).

**Number of excitatory synapses and degree of synchronization**


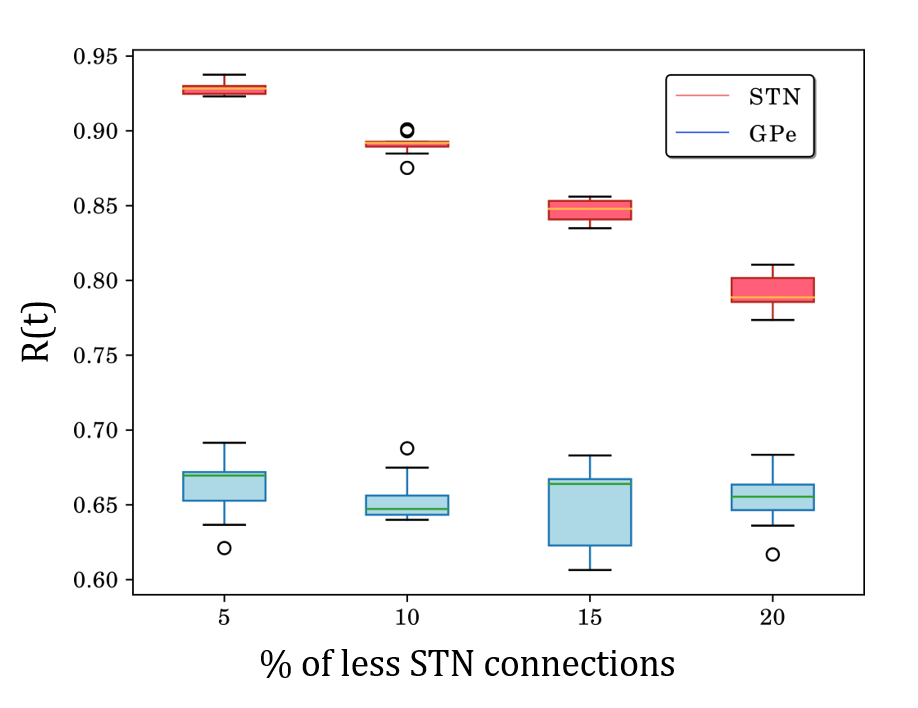


**Supplementary Figure 5. Box plots of order parameters of STN (red) and GPe (blue) after deleting a fraction of STN-STN excitatory synapses, after having the system synchronized with PS, while STDP was active.** Each box plot represents 11 simulations where the model was initialized as in **Figure 3** with different seeds for the random number generator. PS synchronizes the network while STDP is ON, but structural plasticity is permanently OFF. After the PS epoch STDP was turned OFF and a percentage (between 5%-20%) of STN-STN connections was randomly selected with uniform probability and deleted. In the subsequent 2 min time window the amount of synchronization was calculated. The extent of STN synchronization decreases as the percentage of excitatory connections decreases.

**The role of structural plasticity in the brain**

In the brain there are several plasticity rules which take place simultaneously [see e.g. (Engert & Bonhoeffer, 1999; Hübener & Bonhoeffer, 2014; McCann et al., 2008; Song et al., 2000; Turrigiano & Nelson, 2004; van Ooyen & Butz-Ostendorf, 2017; Yuste & Bonhoeffer, 2004; Zenke & Gerstner, 2017)]. The interaction between them, their specific detailed mechanisms and different roles are still not fully understood. However, increasing experimental evidence shows that synaptic plasticity takes place on time scales that may range from seconds to minutes and up to hours [see e.g. (Zenke & Gerstner, 2017)]. In contrast, structural plasticity is a much slower process which has been identified to take place across longer time frames, i.e., from several hours through to days, weeks and months [see e.g. (van Ooyen & Butz-Ostendorf, 2017)]. The homeostatic control was considered as an internal mechanism of neurons which allows them to keep a healthy operating regime (Harnack et al., 2015). The analytical framework in that study was based on control theory and explores possible and adequate conditions for stability in recurrent networks which can be directly linked to different mechanisms of plasticity such as the ones we explore in this study. A review on the recent developments in the field of homeostatic plasticity and the stabilization of neural function can be found in (Davis, 2013). For example, it was shown that during aging the homeostatic signaling system adjusts its response to accommodate to a new set point (Mahoney et al., 2014), a more detailed discussion can be found regarding the existence of many homeostatic points and how these are regulated by certain molecular transporters. In a recent computational study (Lu et al., 2019) a neural network model with structural plasticity enabled to reproduce long-lasting effects of transcranial brain stimulation.

Different types of homeostatic plasticity mechanisms, comprising the adjustment of synaptic strengths or intrinsic excitability were studied in the context of, e.g., activity-dependent development and memory storage (Turrigiano 1999; Turrigiano & Nelson 2004; Zhang & Linden 2003). Perforated patch clamp recordings in rat corticostriatal slices revealed a homeostatic mechanism enabling to maintain firing rates within functional range (Azdad et al., 2009). Specifically, this study showed that dopamine depletion increased the intrinsic excitability of striatal medium spiny neurons by decreasing an inactivating A-type potassium current. The CR-induced change of the target firing rate might be mediated by a mechanism similar to intrinsic plasticity, i.e., to learning-induced changes of the function of voltage-gated ion channels, in turn, leading to sustained changes in the intrinsic excitability of neurons (Zhang & Linden 2003). In Parkinson’s disease several other homeostatic mechanisms may be affected, too, for instance, synaptic homeostasis (Soukup et al., 2018), mitochondrial homeostasis (Ng et al., 2017) and iron homeostasis (Double et al., 2000). In (Lu et al., 2019) the same structural plasticity model used in this manuscript was incorporated in a different neuronal network (without STDP) to model effects of transcranial direct current stimulation. In that study structural plasticity modulated excitatory connections only. Their numerical results were in agreement with empirical evidence related to positive and negative effects of this type of therapy.

**Code and scripts availability**

The NEST version used for the simulations presented in this manuscript can be found here: https://github.com/sdiazpier/nest-simulator.git (branch dbs_sp) and the model scripts can be found in this repository: https://github.com/sdiazpier/dbs_sp.git.

**References**

Azdad, K., Chàvez, M., Bischop, P. D., Wetzelaer, P., Marescau, B., De Deyn, P. P., Gall, D. & Schiffmann, S. N. (2009). Homeostatic Plasticity of Striatal Neurons Intrinsic Excitability following Dopamine Depletion. *PLoS ONE* 4(9): e6908. doi:10.1371/journal.pone.0006908

Davis, G. W. (2013). Homeostatic Signaling and the Stabilization of Neural Function. *Neuron*, *80*(3), 718-728. https://doi.org/10.1016/j.neuron.2013.09.044

Double, K., L., Gerlach, M., Youdim, M. B. H., & Riederer, P. (2000). Impaired iron homeostasis in Parkinson’s disease. *J. Neural. Transm*. [Suppl.] 60:21-42.

Engert, F., & Bonhoeffer, T. (1999). Dendritic spine changes associated with hippocampal long-term synaptic plasticity. *Nature*, *399*, 66. https://doi.org/10.1038/19978

Harnack, D., Pelko, M., Chaillet, A., Chitour, Y., & van Rossum, M. C. W. (2015). Stability of Neuronal Networks with Homeostatic Regulation. *PLOS Computational Biology*, *11*(7), e1004357. https://doi.org/10.1371/journal.pcbi.1004357

Hübener, M., & Bonhoeffer, T. (2014). Neuronal Plasticity: Beyond the Critical Period. *Cell*, *159*(4), 727-737. https://doi.org/10.1016/j.cell.2014.10.035

Lu, H., Gallinaro, J. V., & Rotter, S. (2019). Network remodeling induced by transcranial brain stimulation: A computational model of tDCS-triggered cell assembly formation. *Network Neuroscience*, 1-21. https://doi.org/10.1162/netn_a_00097

Mahoney, R. E., Rawson, J. M., & Eaton, B. A. (2014). An Age-Dependent Change in the Set Point of Synaptic Homeostasis. *The Journal of Neuroscience*, *34*(6), 2111. https://doi.org/10.1523/JNEUROSCI.3556-13.2014

McCann, C. M., Tapia, J. C., Kim, H., Coggan, J. S., & Lichtman, J. W. (2008). Rapid and modifiable neurotransmitter receptor dynamics at a neuronal synapse in vivo [Article]. *Nature Neuroscience*, *11*, 807. https://doi.org/10.1038/nn.2145

Ng, C. H., Hang, L., & Lim, K. L. (2017). Mitochondrial homeostasis in Parkinson’s disease - a triumvirate rule? *Neural Regeneration Research*. 12(8):1270-1272.

Song, S., Miller, K. D., & Abbott, L. F. (2000). Competitive Hebbian learning through spike-timing-dependent synaptic plasticity. *Nature Neuroscience*, *3*, 919. https://doi.org/10.1038/78829

Soukup, S. F., Vanhauwaert, R., & Verstreken, P. (2018). Parkinson’s disease: convergence on synaptic homeostasis. *The EMBO Journal.* 37: e98960

Turrigiano, G. G. (1999). Homeostatic plasticity in neuronal networks: the more things change, the more they stay the same. *Trends Neurosci*. 22: 221–22.

Turrigiano, G. G., & Nelson, S. B. (2004). Homeostatic plasticity in the developing nervous system. *Nature Reviews Neuroscience*, *5*, 97. https://doi.org/10.1038/nrn1327

van Ooyen, A., & Butz-Ostendorf, M. (2017). *The Rewiring Brain A Computational Approach to Structural Plasticity in the Adult Brain*. Academic Press

Yuste, R., & Bonhoeffer, T. (2004). Genesis of dendritic spines: insights from ultrastructural and imaging studies [Review Article]. *Nature Reviews Neuroscience*, *5*, 24. https://doi.org/10.1038/nrn1300

Zenke, F., & Gerstner, W. (2017). Hebbian plasticity requires compensatory processes on multiple timescales. *Philosophical transactions of the Royal Society of London. Series B, Biological sciences*, *372*(1715), 20160259. https://doi.org/10.1098/rstb.2016.0259

Zhang, W., & Linden, D. J. (2003). The other side of the engram: experience-driven changes in neuronal intrinsic excitability. *Nature Rev. Neurosci*. 4: 885-900.
